# Selenoprotein M stimulates the proliferative and metastatic capacities of renal cell carcinoma through activating the PI3K/AKT/mTOR pathway

**DOI:** 10.1002/cam4.2403

**Published:** 2019-07-05

**Authors:** Hao Jiang, Qian‐Qian Shi, Li‐Yuan Ge, Qian‐Feng Zhuang, Dong Xue, Hai‐Yan Xu, Xiao‐Zhou He

**Affiliations:** ^1^ Department of Urology The Third Affiliated Hospital of Soochow University Changzhou People's Republic of China; ^2^ Department of Urology Peking University Third Hospital Beijing People's Republic of China

**Keywords:** migration and invasion, PI3K/Akt/mTOR, proliferation, renal cell carcinoma, Selenoprotein M

## Abstract

High‐throughput sequencing methods have facilitated the identification of novel selenoproteins, which exert a vital role in the development and progression of tumor diseases. Recently, Selenoprotein M (SELM) is upregulated in several types of cancer. However, the biological roles of SELM in renal cell carcinoma (RCC) remain unclear. In this paper, quantitative reverse transcription PCR (qRT‐PCR) and Western blot were used to measure relative levels of SELM in a cohort of RCC tissues with matched normal tissues as well as human RCC cell lines. SELM expression was found to be upregulated in RCC. High level of SELM was related to poor prognosis of RCC. Furthermore, silence of SELM could inhibit the in vitro proliferative, migratory, and invasive capacities of RCC. In addition, downregulated SELM could impede in vivo tumorigenesis of RCC. SELM could activate the PI3K/Akt/mTOR pathway and mediate expressions of matrix metallopeptidase 2 and 9 (MMP2, MMP9). In conclusion, our study reveals the oncogenic function of SELM in RCC, and SELM may be a therapeutic and prognostic target for RCC.

## 1 INTRODUCTION

The mortality and morbidity of renal cell carcinoma (RCC), a prevalent urological tumor, have been steadily risen.^1^ Up to 80% of primary renal neoplasms are clear cell renal cell carcinoma (ccRcc).^2^ About 20%‐40% of patients suffer from metastatic foci at the initial diagnosis.^3^ Seriously, the 5‐year survival of RCC is lower than 55%.^4^, ^5^ Despite the advanced inspection technologies and therapeutic strategies (ie, surgery, radiotherapy, and molecular targeted therapy) are extensively applied, the prognosis of RCC remains poor.^3^, ^6^ Although many potential molecular therapeutic biomarkers have been identified in RCC, the accurate mechanism of RCC pathogenesis and progression is still incompletely understood.^7^ Therefore, it is urgently needed to uncover the pathogenic mechanism of RCC and develop reliable targets for RCC treatment.

As an essential trace element, selenium is of significance in human health.^8^ Epidemiologic, preclinical, and clinical studies have uncovered that selenium may inhibit the malignant growth of RCC through mediating selenoproteins.^9^ Selenoprotein M (SELM), a kind of selenoproteins located at the membranes of the cellular endoplasmic reticulum (ER), has been well concerned owing to its unique redox motif of cysteine‐X‐X‐selenocysteine compared with other selenoproteins.^10^ Recently, a growing number of evidence has revealed that selenoprotein participates in multiple biological process, including cellular behaviors,^11^ anti‐antioxidant,^11^ neuroprotective properties,^12^ anti‐ER stress,^13^ and cytosolic calcium regulation.^14^

Both SELM and the 15‐kDa selenoprotein (Sep15) belong to a unique selenoprotein family, which has an NH2‐terminal signal peptide and a thioredoxin‐like domain. Previous studies have reported that Sep15 participates in regulating tumorigenesis and the progression of cancers, including liver,^15^ breast,^16^ prostate,^17^ and lung cancers.^18^ The specific function of SELM in RCC, however, is unclear. In this study, expression pattern and biological function of SELM in RCC were mainly investigated.

## 2 MATERIALS AND METHODS

### 2.1 Sample and data collection

Renal tumor tissues and pericarcinous tissues were surgically resected from RCC patients admitted at the Department of Urology, the Third Affiliated Hospital of Soochow University, from February 2009 to August 2012. The follow‐up deadline was November 2017. RCC patients were diagnosed according to World Health Organization classification. The cancer samples were divided into I‐IV stages according to the Fuhrman histologic grading system. This study got approval by the Institutional Research Ethics Committee of the Third Affiliated Hospital of Soochow University.

### 2.2 Quantitative reverse transcription PCR

Total RNA extraction was performed with TRIzol reagent (Takara, Otsu, Japan). Subsequently, RNA was reversely transcribed using the PrimeScript RT Reagent Kit (Takara, Otsu, Japan). The synthesized cDNA underwent quantitative reverse transcription PCR (qRT‐PCR) using SYBR green (Takara) on the Applied Biosystems 7500 Real‐Time PCR System (Foster City, CA). Relative levels of genes were calculated by 2^−△△CT^ that normalized to the expression of GAPDH (glyceraldehyde 3‐phosphate dehydrogenase). The sequence of primers were as follows: Selenoprotein M, (forward: GAACCGTCTGAGCGGCCTAA, reverse: GGAGGTGTTTCATCACCAGGTTG) and GAPDH (forward: GAGAGACCCTCACTGCTG, reverse: GATGGTACATGACAAGGTGC).

### 2.3 Western blot

Cell lysis was prepared on ice and subjected to centrifugation at 4°C, 14 000 *g*/min for 10 minutes. Proteins were quantified using a BCA Protein Assay Kit (Sigma‐Aldrich) and separated by 10% SDS‐PAGE gel. Subsequently, proteins were loaded on a PVDF membrane (Millipore, Billerica, MA), and incubated with specific antibodies. Antibodies were purchased from Abcam (SELM, PI3K, phosphor‐PI3K, mTOR, phosphor‐mTOR, vimentin, N‐cadherin, and β‐cadherin), Cell Signaling Technology (Akt, phosphor‐Akt, MMP2, MMP9, anti‐rabbit, and anti‐mouse secondary antibodies), and Arigo (GAPDH).

### 2.4 Cell culture

Renal cell carcinoma cell lines (786O, 769P, ACHN, and CAKI‐1) and the normal human epithelial cells of renal tubules (HK2) were provided by the American Type Culture Collection (ATCC, Manassas, VA) and Cell Biology of the Chinese Academy of Sciences (Shanghai, China). Except for CAKI‐1 cells cultured in McCoy's 5A medium, the remaining were cultured in RPMI‐1640 medium (GIBCO, Carlsbad, USA) containing 10% fetal bovine serum (FBS, GIBCO) and 1% penicillin/streptomycin (Invitrogen). Cells were maintained in a humidified environment at 37°C with 5% CO_2_. The phosphatidylinositol 3‐kinase (PI3K) inhibitor LY294002 was obtained from Selleck Chemical (Houston, TX) (no.S1105).

### 2.5 Transfection

Lentiviral vectors phU6‐EGFP‐shRNA‐SELM, pUbi‐EGFP‐SELM, and their controls were prepared by GeneChem Co., Ltd. (Shanghai, China). Transfection in CAKI‐1 and 786O cells was conducted following the manufacturer's recommendations.

### 2.6 Cell proliferation assays

Cells were inoculated into 96‐well plates with 3000 cells/well. After cell culture for 24, 48, 72, and 96 hours, CCK‐8 (Cell Counting Kit‐8, Dojindo Laboratories, Kumanmoto, Japan) solution was applied. After 2 hours, the absorbance at 450 nm was recorded using a microplate reader. For the colony formation assay, cells were inoculated in 6‐well plates with 1000 cells/well. After 14 days, the colonies were subjected to methanol fixation and 0.1% crystal violet (Sigma‐Aldrich) staining. Visible colonies were counted. The experiment was performed in triplicate.

### 2.7 Transwell assay

24‐well Transwell chambers (8 μm diameter, Costar, Corning, NY) precoated either with Matrigel (Invitrogen) or not were used (Matrigel precoating was necessary in the Transwell invasion assay). Briefly, 2 × 10^4^ cells suspended in 200 µL of serum‐free medium were applied on the upper chambers. Complete medium was applied on the bottom. After 24‐hour cell culture, penetrated cells on the bottom were dyed with 0.1% crystal violet. Migratory and invasive cell numbers were counted in five randomly selected fields. The final data were recorded from three individual experiments.

### 2.8 In vivo tumorigenesis assay

Female nude mice of 5‐week‐old underwent subcutaneous injection of shSELM‐Caki‐1 stably expressed cells (7 × 10^6^) and control cells (NC‐Caki‐1) suspended in 150 µL of PBS at the single side of the posterior flank. Tumor size was measured once a week. The tumor volume was: V = length × width^2^ × 0.52 (V, volume; length, longitudinal diameter; and width, latitudinal diameter of the tumor). Six weeks later, tumors were harvested, weighed, and prepared for immunohistochemistry (IHC). This study followed the guidances for the Care and Use of Laboratory Animals of the National Institutes of Health and the Animal Research Ethics Committee of Soochow University.

### 2.9 IHC

SELM‐positive level in tissues was evaluated by IHC. The tissue paraffin sections were incubated with the primary antibody at 4°C overnight and HRP‐conjugated secondary antibody, followed by diaminobenzidine dyeing. IHC results were evaluated by two experienced pathologists. RCC patients were assigned into low‐ and high‐staining groups for further analyses.

### 2.10 Statistical analysis

SPSS 22.0 software was used for data processing. All of the data were presented as mean ± SD from three records. The results were analyzed using the student's *t* test and Chi‐squared test. Kaplan‐Meier method was introduced for survival analysis. *P* < 0.05 considered as statistically significant.

## 3 RESULTS

### 3.1 Upregulated SELM in RCC

**Figure 1:**
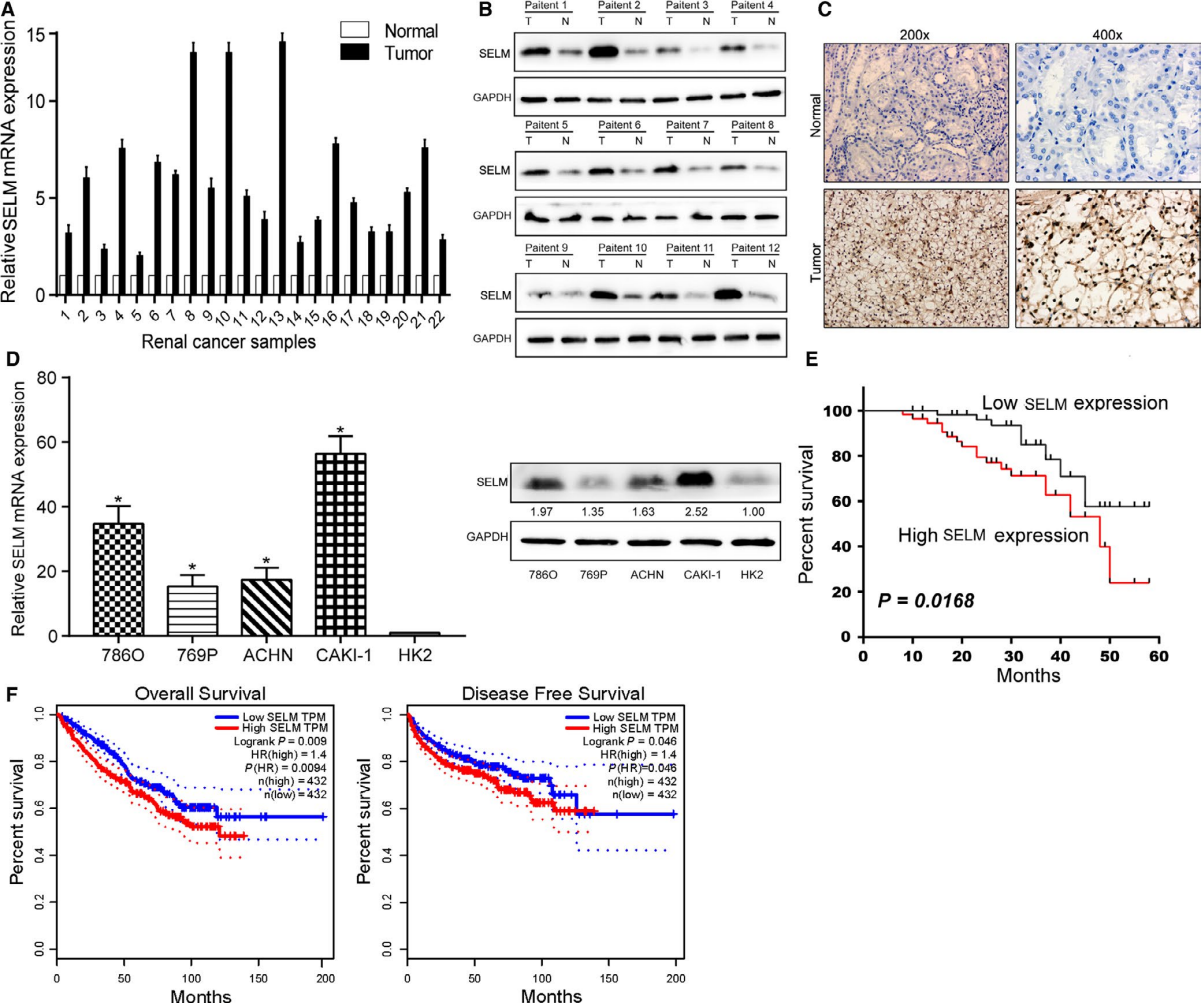
Selenoprotein M (SELM) is upregulated in renal cell carcinoma (RCC) and associated with poor prognosis. (A), the relative expression of SELM was examined by qRT‐PCR in 22 pairs of RCC tissues and paired normal tissues; (B), the relative protein levels of SELM were examined by Western blot in 22 pairs of RCC tissues and paired normal tissues; (C), the relative protein levels of SELM were examined by immunohistochemistry in 125 pairs of RCC tissues and normal tissues; (D), the relative expression of SELM was examined by qRT‐PCR and Western blot in RCC cell lines (786O, 769P, ACHN, and CAKI‐1) and a normal epithelium cell line of renal tubules (HK2); E and F, Kaplan‐Meier survival curves of patients with RCC based on SELM expression. Patients in the high expression had a markedly more unfavorable prognosis than those in low expression group (*P* = 0.0168, log‐rank test). *P < 0.05, **P < 0.01

Both mRNA and protein levels of SELM were markedly upregulated in 22 RCC tissues relative to the adjacent ones (Figure 1A,B). Meanwhile, IHC results obtained in a cohort of 125 paired RCC tissues also revealed the upregulated SELM in RCC (Figure 1C). Moreover, in vitro level of SELM was highly expressed in RCC cells relative to HK2 (Figure 1D). It is suggested that SELM may be a potential biomarker in the progression of RCC.

### 3.2 SELM is correlated with prognosis of RCC

The correlation between SELM expression and pathological characteristics of RCC was assessed. According to the median level of SELM, 125 RCC patients were assigned into high‐SELM level group (n = 66) and low‐SELM level group (n = 59). SELM level was positively correlated to histological grade (*P* = 0.019) and tumor node metastasis (TNM) staging (*P* = 0.025), rather than age (*P* = 0.915), gender (*P* = 0.826), tumor size (*P* = 0.679), and tumor histology (*P* = 0.602) of RCC patients (Table 1). Moreover, survival analysis showed that high level of SELM predicted shorter overall survival in RCC patients (Figure 1E). Similarly, analyses of the TCGA data also identified a worse prognosis in RCC patients expressing high‐level SELM (Figure 1F). Overall, SELM may aggravate the progression of RCC.

**Table 1:** Association of Selenoprotein M (SELM) expression with clinicopathologic characteristics of renal cell carcinoma patients

Parameters	Number of cases	SELM expression		*P*‐value
		Low	High	
Age (years)				
≤60	79	37	42	0.915
>60	46	22	24	
Gender				
Male	75	36	39	0.826
Female	50	23	27	
Tumor size (cm)				
≤4	59	29	30	0.679
>4	66	30	36	
Histology				
Clear cell carcinoma	116	54	62	0.602
Others	9	5	4	
Histological grade				
I‐II	104	54	50	0.019
III‐IV	21	5	16	
TNM stage				
I	97	51	46	0.025
II‐IV	28	8	20	

### 3.3 SELM regulates in vitro viability of RCC cells

**Figure 2:**
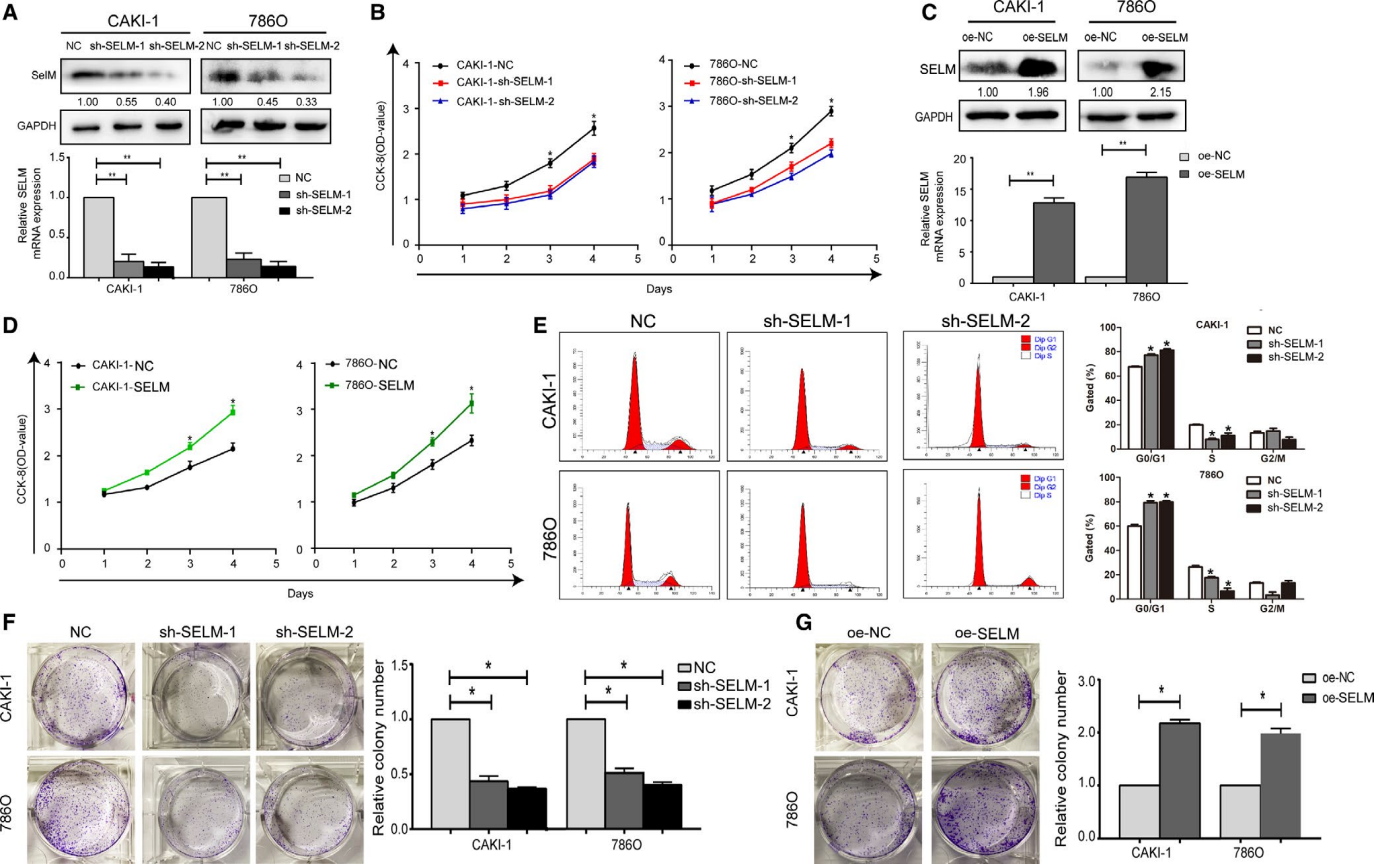
The effects of Selenoprotein M (SELM) on renal cell carcinoma (RCC) cell proliferation and growth in vitro. (A), The relative expression of SELM in CAKI‐1 and 786O cells was detected by Western blotting and qRT‐PCR; (B) Growth curves show the cell growth of CAKI‐1 and 786O cells under SELM knockdown by CCK‐8 assay; (C) The relative expression of SELM in CAKI‐1 and 786O cells was detected by Western blotting and qRT‐PCR; (D) Growth curves show the cell growth of CAKI‐1 and 786O cells under SELM overexpression by CCK‐8 assay; (E), The cell cycle progression of CAKI‐1 and 786O cells under SELM knockdown was detected by Flow cytometry; (F and G) The efficiency of cell colony formation in CAKI‐1 and 786O cells with SELM knockdown or overexpression was evaluated by colony formation assay

CAKI‐1 and 786O cells expressing high abundance of SELM were transfected with phU6‐EGFP‐shRNA‐SELM or pUbi‐EGFP‐SELM. Transfection of phU6‐EGFP‐shRNA‐SELM sufficiently downregulated SELM, and conversely, transfection of pUbi‐EGFP‐SELM upregulated SELM level in CAKI‐1 and 786O cells (Figure 2A). CCK‐8 assay revealed that silence of SELM decreased the viability in CAKI‐1 and 786O cells (Figure 2B). On the contrary, overexpression of SELM enhanced cell viability (Figure 2C‐D). Furthermore, flow cytometry analysis showed that downregulation of SELM resulted in increased cell ratio in G1 and decreased one in S phase (Figure 2E). Consistently, colony formation assay showed that knockdown of SELM suppressed colony formation capacity of CAKI‐1 and 786O cells (Figure 2F‐G).

### 3.4 SELM regulates the metastasis of RCC cells by influencing epithelial‐mesenchymal transition

**Figure 3:**
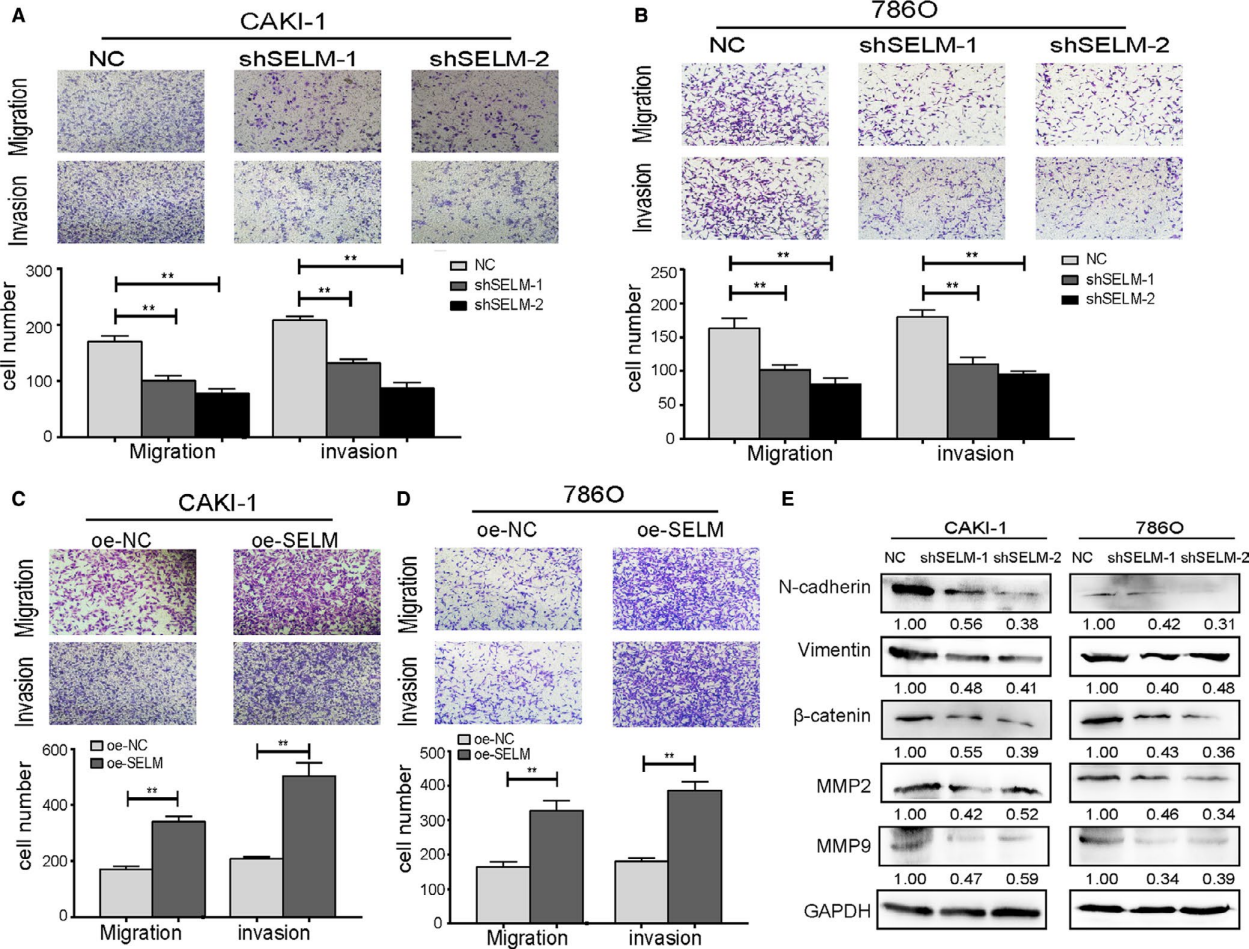
Selenoprotein M (SELM) promotes cell migratory and invasive ability in vitro. (A‐D), Transwell assays were used to evaluate the migration and invasion ability of CAKI‐1 and 786O cells with SELM knockdown or overexpression; (E) The protein levels of N‐cadherin, vimentin, β‐catenin, MMP2, and MMP9 were analyzed by Western blotting in CAKI‐1 and 786O cells with SELM knockdown or overexpression. Data represent the mean ± SD from three independent experiments. **P < 0.01

Transwell assay showed that downregulation of SELM attenuated the migratory and invasive abilities of CAKI‐1 and 786O cells, while overexpression of SELM achieved the opposite trends (Figure 3A‐D). Epithelial‐mesenchymal transition (EMT) is generally considered to be related with tumor cell invasion and metastasis. Here, Western blot was performed to assess EMT‐related gene expressions influenced by SELM in RCC cells. N‐cadherin, Vimentin, β‐catenin, MMP2, and MMP9 were remarkably downregulated by silence of SELM (Figure 3E). Taken together, these data indicated that SELM could affect the metastatic ability of RCC cells by stimulating EMT.

### 3.5 SELM affects the PI3K/AKT/mTOR pathway in RCC

**Figure 4:**
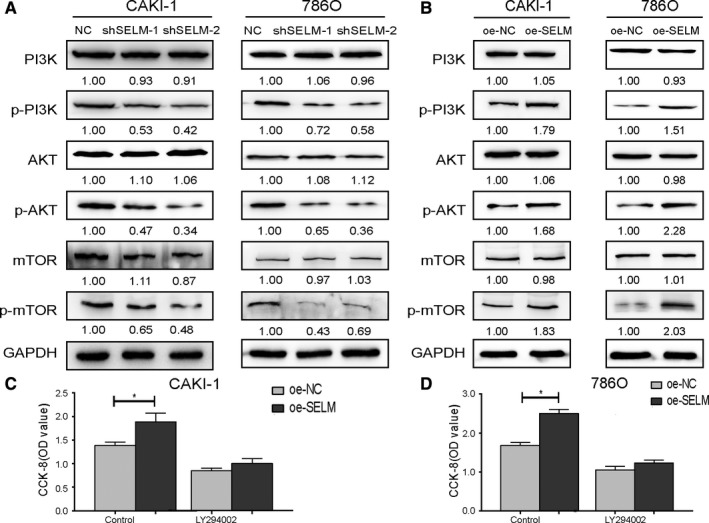
The effect of Selenoprotein M (SELM) on mTOR/AKT pathway. (A and B), The protein expression levels of PI3K, phosphor‐PI3K, AKT, phosphor‐AKT, mTOR, and phosphor‐mTOR were detected by Western blotting in CAKI‐1 and 786O cells with SELM knockdown or overexpression; (C and D) The proliferation capacity of CAKI‐1 and 786O with SELM overexpression (SELM) and control cells (NC) was detected by CCK‐8 assay after treatment with LY294002 for 48 h. Data represent the mean ± SD from three independent experiments, *P < 0.05

Subsequently, Western blot analyses revealed that p‐PI3K, p‐AKT, and p‐mTOR were downregulated in RCC cells with SELM knockdown. RCC cells overexpressing SELM presented the opposite trends. Nevertheless, the total expressions of PI3K, AKT, and mTOR were not altered by SELM (Figure 4A‐B). Furthermore, a PI3K inhibitor (LY294002, 20 μmol/L) was applied to validate our results. LY294002 treatment for 48 hours reversed the regulatory effects of SELM on the proliferative ability of RCC (Figure 4C‐D).

### 3.6 Silence of SELM inhibits tumorigenesis of RCC in vivo

**Figure 5:**
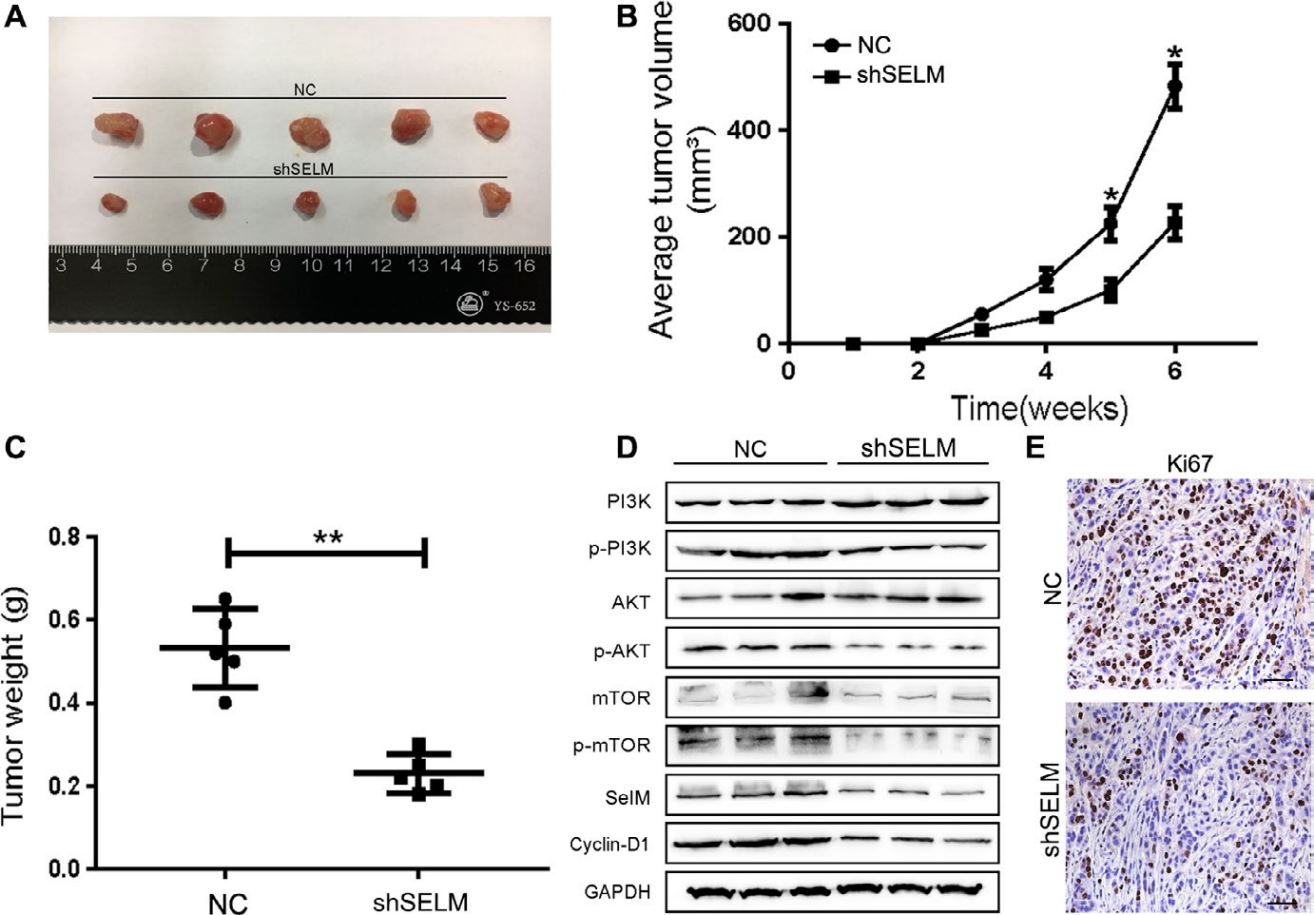
Knockdown of Selenoprotein M (SELM) impedes tumorigenesis in vivo. (A), Representative pictures of tumor in CAKI‐1‐NC and CAKI‐1‐shSELM cell‐transplanted mice; (B) Tumor volume was calculated at the indicated weeks after mice were transplanted; (C) Tumor weight was measured after tumor removal; (D) The protein expression levels of PI3K, phosphor‐PI3K, AKT, phosphor‐AKT, mTOR, and phosphor‐mTOR etc were detected by Western blotting in tumor tissues from CAKI‐1‐NC and CAKI‐1‐shSELM cell‐transplanted mice; (E) Ki‐67 immunohistochemistry was performed to evaluate cell proliferation in tumor tissues from CAKI‐1‐NC and CAKI‐1‐shSELM cell‐transplanted mice. *P < 0.05, **P < 0.01

A nude mouse xenograft model was constructed to identify the in vivo effect of SELM on RCC. Subcutaneous injection of shSELM‐Caki‐1 stably expressed cells (7 × 10^6^) and control cells (NC‐Caki‐1) were administrated (Figure 5A). The tumor volume of the SELM silence group (shSELM) was markedly smaller than that of controls since the fifth week (Figure 5B). Lower tumor weight was also observed in shSELM group (Figure 5C). Additionally, inhibition of SELM consistently suppressed Ki67 expression, a proliferation marker, in CAKI‐1 xenograft tumors (Figure 5D‐E). Collectively, these results further revealed that silence of SELM could markedly inhibit tumorigenesis in vivo.

## 4 DISCUSSION

RCC is the most prevalent kidney cancer with a high mortality and morbidity in China, and its poor prognosis severely influences the affected patients.^19^, ^20^, ^21^ Accumulating evidences have demonstrated various tumor‐related genes in the tumorigenesis of RCC. Recently, selenoproteins were found to be involved in tumor progression.^22^, ^23^ SELM, a novel selenoprotein, is a thioredoxin‐like fold ER‐resident protein. It is upregulated in hepatocellular carcinoma.^24^ However, its potential function in RCC remains unclear. In this paper, SELM was upregulated in RCC, and correlated with higher histological stage, advanced TNM stage, and shorter overall survival of RCC patients. Silence of SELM markedly reduced the viability, clonality, and metastasis of RCC cells. Furthermore, a tumor xenograft mouse model demonstrated that downregulation of SELM could significantly restrain the in vivo growth of tumors. Overall, these data suggested that SELM served as an oncogenic role in RCC.

The beneficial influence of selenium could be attributed to its presence within selenoproteins, which play crucial roles in multiple tumors. Guerriero et al^24^ demonstrated that SELM is overexpressed in human hepatocellular carcinoma tissues and its level is positively correlated to the malignant level. Hwang et al^25^ reported that overexpression of SELM in CMV/GFP‐hSELM rats enhances antioxidant enzyme activities, which are important for the regulation of tumorigenesis and tumor progression. Furthermore, Reeves et al^11^ found that SELM is implicated in cytosolic calcium regulation. Silence of SELM results in increased baseline levels of cytosolic calcium and thus leads to apoptosis. This study illustrated the oncogenic role of SELM in RCC via regulating in vitro and in vivo proliferation and metastasis. Uncontrolled proliferation of tumor cells is a basic characteristics of carcinogenesis that could be affected by the PI3K/Akt/mTOR pathway.^26^ This pathway is of significance in targeted therapy, which is considered as checkpoints for growth stimuli. Moreover, the activated Akt/mTOR pathway results in drug resistance in tumors, leading to an unsatisfactory outcome.^27^ Our study demonstrated that overexpression of SELM activated the PI3K/Akt/mTOR pathway. Additionally, LY294002 application could reverse the promotive effect of SELM on proliferative and metastatic abilities of RCC cells.

Migratory and invasive progressions are two major events in tumor metastasis.^28^ Over the past decade, EMT has been found to participate in metastasis by influencing cancer cell motility and dissemination.^29^ N‐cadherin and vimentin are generally used as mesenchymal markers for EMT.^30^ In addition, MMPs are positively related to tumor progression, metastasis, and prognosis of cancers.^31^, ^32^ Our study found that N‐cadherin, β‐catenin, vimentin, and MMPs were markedly upregulated in RCC cells overexpressing SELM. Therefore, we considered that SELM may facilitate the metastasis of RCC by regulating EMT and extracellular matrix degradation.

In conclusion, our study suggested that SELM was upregulated in RCC. SELM served as an oncogene in RCC via activating the PI3K/Akt/mTOR pathway and EMT. This study provides a theoretical basis that SELM may be a potential target for predicting the prognosis of RCC.

## CONFLICT OF INTEREST

None declared.

## Data Availability

The data that support the findings of this study are available from the corresponding author upon reasonable request.
